# Paxlovid-Induced Symptomatic Bradycardia and Syncope

**DOI:** 10.7759/cureus.33831

**Published:** 2023-01-16

**Authors:** Venu M Ganipisetti, Pratyusha Bollimunta, Sriram Maringanti

**Affiliations:** 1 Hospital Medicine, Presbyterian Hospital, Albuquerque, USA; 2 Hospital Medicine, University of New Mexico, Albuquerque, USA

**Keywords:** covid-19, protease inhibitor, recurrent syncope, bradycardia, nirmatrelvir, ritonavir, cardioinhibitory syncope, drug induced bradycardia, covid 19 treatment, paxlovid

## Abstract

Paxlovid (nirmatrelvir/ritonavir) is a game changer in the fight against COVID-19 due to its ease of administration and significant benefits of reducing progression to severe COVID-19, hospitalization, and death. Cardiac adverse events such as bradycardia and syncope are not known with this medication. We report a case of a 71-year-old patient who developed symptomatic bradycardia, syncopal episodes, and sinus pause after taking Paxlovid. Discontinuing medication and intravenous atropine helped to reverse the bradycardia and symptoms promptly. She did not require a pacemaker. We would like to report this possible association between Paxlovid and bradycardia. Until further information or studies are available, it is advised to promptly discontinue Paxlovid after any evidence of bradycardia and closely monitor for at least 40 hours in a hospital setting. The reported half-life (t 1/2) of the medication is 6.05 ± 1.79 hours and using 8 hours as a reference for the upper limit of t 1/2, around 97 % of the medication should be cleared off in about 40 hours (five half-lives).

## Introduction

COVID-19 infection, caused by severe acute respiratory syndrome coronavirus 2 (SARS-CoV-2) (a novel coronavirus), which started as a small outbreak in Wuhan, China in 2019 has become a global pandemic. According to the WHO, as of January 6, 2023, there are 657 million reported cases of COVID-19 and resulting in estimated 6.68 million deaths worldwide [1].

Infection is divided into symptomatic and asymptomatic cases. Symptomatic cases are further categorized into non-severe (mild and moderate), severe, and critical cases [2]. Factors that increase the risk of severe disease include age ≥65 years, comorbidities such as asthma, chronic kidney disease, chronic liver disease, cardiovascular disease, diabetes, HIV (untreated or advanced), obesity, pregnancy, cigarette smoking, immunosuppressive therapy, or transplant status [3].

Multiple treatment options have been authorized for use in the outpatient treatment of mild or moderate COVID-19. These include Paxlovid (nirmatrelvir/ritonavir), remdesivir, and molnupiravir [4]. Paxlovid and molnupiravir are administered in an oral form and can be more conveniently taken at home, whereas remdesivir must be administered intravenously.

Paxlovid is authorized for emergency use by the FDA for mild and moderate cases in age groups 12 years and older, with risk factors for severe disease, and should be started within five days of symptom onset [5]. It has been shown to reduce the risk of progression to severe disease, hospitalization, and death with a relative risk reduction rate of 89% [6].

We report a case of a 71-year-old female, who had acquired mild COVID-19 infection and was prescribed Paxlovid due to increased risk for severe disease with her age. Subsequently, she developed syncopal episodes at home and was hospitalized for evaluation. She had additional episodes of symptomatic bradycardia and sinus pause, which were successfully reversed with IV atropine. The patient did not require a pacemaker. When used alone, Paxlovid was not reported to cause cardiac adverse events such as bradycardia, sinus dysfunction, or syncope. This is probably the first case reporting this association.

## Case presentation

A 71-year-old female presented to our emergency room (ER) with two syncopal episodes. About three days prior to the presentation, she started having a fever, dry cough, body aches, and tested positive for COVID-19 at home. She was started on a 5-day treatment course of Paxlovid (nirmatrelvir/ritonavir). Subsequently, the patient developed episodes of nausea with vomiting. After the third dose of Paxlovid, she had sudden onset of dizziness with nausea and felt like she was about to pass out. She was able to slowly bring herself to the ground and then lost consciousness. This was an unwitnessed episode, and her family members noticed her on the ground about 10 minutes later. The patient was able to stand up, walked to the bed, and then lost consciousness again for 3-5 minutes. No seizure-like activity was noted. She presented to ER after the second syncopal episode.

She has no significant past medical history apart from the remote history of vasovagal syncopal episodes. Her last syncopal episode from vasovagal episodes was more than 30 years ago. She does not drink alcohol, smoke cigarettes, or use any recreational drugs. She is not on any other prescription medications.

An initial evaluation in the ER was reassuring. Her blood pressure (BP) was 143/87 mmHg, temperature 98.4°F, heart rate 62 beats per minute, and respiratory rate 16 per minute. Labs showed normal electrolytes, and renal and liver function. D-dimer was negative. Orthostatic vitals were unremarkable. High-sensitivity serial troponins were negative. EKG showed normal sinus rhythm with heart rate of 64, normal QRS wave, good R wave progression with no Q waves or significant ST-T abnormalities. She remained asymptomatic and appeared stable for discharge from the ER. While preparing for discharge, she suddenly developed lightheadedness, nausea with vomiting, and heart rate suddenly dropped to 28. BP was unrecordable. She was not on a cardiac monitor as patient was about to leave the hospital. She was given a dose of 1 mg IV atropine push with which her heart rate improved to 80s instantly and her BP improved to 141/79 mmHg. She was admitted to the hospital.

**Figure 1:**
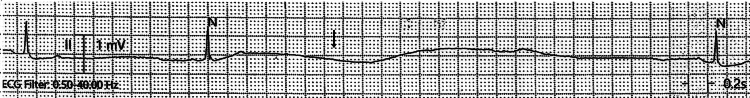
5.28-second pause on telemetry.

**Figure 2:**
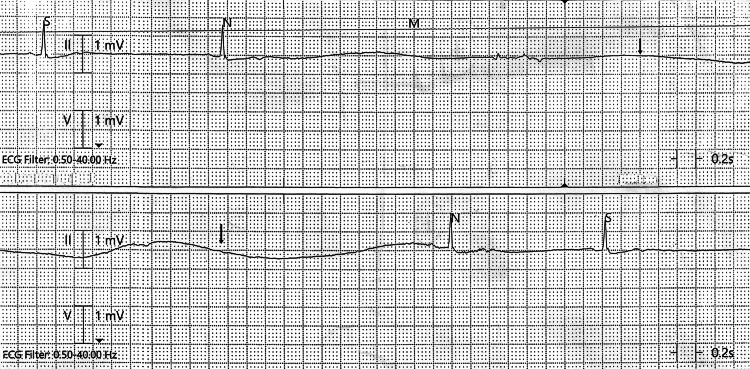
10-second pause on telemetry.

While monitoring, around the 30-hour mark since the last dose of Paxlovid, she developed an episode of sudden lethargy and passed out. She was noted to have several pauses with a maximum 10-second pause on telemetry (Figures 1-2) and was hypotensive. She was given 1 mg IV atropine push, with which her heart rate immediately restored to the 80s. Mild hypokalemia (3.4 mmol/L; reference range 3.5-5.1 mmol/L) was noted and corrected. Her heart rate remained stable in normal sinus rhythm without any further episodes or significant alarms on telemetry. 2D echocardiogram did not show any abnormal findings such as pericardial effusion or valvular abnormalities. The ejection fraction was 74%. The atrial size was normal and no evidence of pulmonary hypertension was noted. Thyroid studies were within normal range. She was monitored for a total of 72 hours in the hospital and no significant cardiac events were noted after the 30-hour mark of Paxlovid.

The cardiac electrophysiology team was consulted. Since these events happened after the initiation of Paxlovid and improved after discontinuation of medication, it was felt that this is a medication-related side effect. Hence, a pacemaker was deferred and she was sent home on a 14-day event monitor for further monitoring. A follow-up phone call was made to her 12 days after her discharge and she reported no further episodes of lethargy or syncope and was feeling much better.

## Discussion

Paxlovid is an oral treatment option for mild to moderate COVID-19 cases, authorized by FDA for emergency use in 2022. It is co-packaged with two drugs, nirmatrelvir 150 mg and ritonavir 100 mg in tablet form. Nirmatrelvir is a protease inhibitor, active against Mpro that plays an important role in viral replication, thereby stopping viral infectivity. Ritonavir enhances the efficacy of nirmatrelvir due to its strong cytochrome P4503A4 (CYP3A4) inhibition properties. Indications for this treatment are patients with mild to moderate COVID-19 (12 years of age and older), who are at risk of developing severe COVID-19 due to risk factors [5]. Studies showed a significant benefit of Paxlovid in reducing the risk of hospitalization and death (89% relative risk reduction in unvaccinated people) [6]. It continues to show good efficacy in reducing severe COVID-19 and mortality, even in the omicron era [7,8]. It is administered in a pill form and can be conveniently used at home, whereas remdesivir, which has almost a similar efficacy rate (87% relative reduction rate), must be given as an infusion in specialized settings [9]. Paxlovid also appears to have greater efficacy compared to the other oral treatment option, molnupiravir (50% relative reduction) [10].

The usual side effects of Paxlovid include allergic reactions, high BP, diarrhea, nausea, abdominal pain, dyspnea, altered sense of taste, feeling generally unwell, muscle aches, liver problems, etc. [5]. Upon literature search, we did not find any reports of Paxlovid (nirmatrelvir-ritonavir) causing bradycardia/sinus dysfunction/syncope, especially when this medication was used alone. However, there were previous reports of bradycardia with ritonavir-based combinations such as lopinavir-ritonavir, etc. The exact pathophysiology is unclear. It is possible that the ritonavir component has contributed to her presentation. Being a strong CYP3A4 inhibitor, ritonavir can increase the risk of cardiotoxicity when used concomitantly with certain medications. However, our patient was not on any medications other than Paxlovid. To our knowledge, this is the first reported case of syncope and bradycardia with sinus dysfunction secondary to nirmatrelvir-ritonavir. Awareness of possible side effects of bradycardia/sinus pause/syncope with Paxlovid is essential until further studies to confirm the mechanism of action and frequency of association are available.

Our patient also has had a history of vasovagal syncope but hasn’t had a single syncopal episode for more than 30 years. Extra caution may be required in prescribing Paxlovid to patients with vasovagal syncopal tendency until further information is available.

Half-life elimination of Paxlovid is reported to be 6.05 ± 1.79 hours [5]. It is well established that 94-97% of a drug is usually eliminated from the body within four to five half-lives [11]. For Paxlovid, five half-lives would be 40 hours (calculated using 8 hours as the maximum reported half-life). Our patient’s last noted cardiac event was at the 30-hour mark since the last dose. These events were fortunately reversed with the administration of intravenous atropine and did not recur. Our patient did not require pacemaker placement.

## Conclusions

We hereby report a potential association between Paxlovid use and sinus node dysfunction. More data is needed to support this association, until then caution may be advised while using Paxlovid, especially in patients with a history of vasovagal syncope. If patients experience sinus node dysfunction, bradycardia, or syncope, withholding the medication may resolve the symptoms and can potentially avoid the placement of a permanent pacemaker. These patients should be monitored in a hospital setting with emergency protocols in place for at least 40 hours after the last dose of Paxlovid.
